# Immune Cells Profiling in ANCA-Associated Vasculitis Patients—Relation to Disease Activity

**DOI:** 10.3390/cells10071773

**Published:** 2021-07-13

**Authors:** Marcelina Żabińska, Katarzyna Kościelska-Kasprzak, Joanna Krajewska, Dorota Bartoszek, Hanna Augustyniak-Bartosik, Magdalena Krajewska

**Affiliations:** 1 Department of Nephrology and Transplantation Medicine, Faculty of Medicine, Wroclaw Medical University, 50-367 Wrocław, Poland; katarzyna.koscielska-kasprzak@umed.wroc.pl (K.K.-K.); dorota.bartoszek@umed.wroc.pl (D.B.); hanna.augustyniak-bartosik@umed.wroc.pl (H.A.-B.); magdalena.krajewska@umed.wroc.pl (M.K.); 2 Department of Otolaryngology, Head and Neck Surgery, Faculty of Medicine, Wroclaw Medical University, 50-367 Wrocław, Poland; j.krajewska@umed.wroc.pl

**Keywords:** ANCA-associated vasculitides, immunophenotyping, flow cytometry

## Abstract

Antineutrophil cytoplasmic antibody (ANCA)-associated vasculitides (AAV) are a group of necrotizing multiorgan autoimmune vasculitides that predominantly affect small blood vessels and are associated with the presence of ANCAs. The aim was to assess regulatory and effector cell populations accompanied by the suPAR biomarker level and link the so-defined immune state to the AAV disease activity. The research involved a multicomponent description of an immune state encompassing a range of B and T cell subsets such as transitional/regulatory B cells (CD19^+^CD24^++^CD38^++^), naïve B cells (CD19^+^CD24^INT^CD38^INT^), Th17 cells, T regulatory cells (CD4^+^CD25^+^FoxP3^+^) and cytotoxic CD4^+^CD28^−^ cells by flow cytometry. The suPAR plasma level was measured by ELISA. The results indicate that AAV is associated with an increased suPAR plasma level and immune fingerprint characterized by an expansion of Th17 cells and T cells lacking the costimulatory molecule CD28, accompanied by a decrease of regulatory populations (Tregs and transitional B cells) and NK cells. Decreased numbers of regulatory T cells and transitional B cells were shown to be linked to activation of the AAV disease while the increased suPAR plasma level—to AAV-related deterioration of kidney function. The observed immune fingerprint might be a reflection of peripheral tolerance failure responsible for development and progression of ANCA-associated vasculitides.

## 1. Introduction

Antineutrophil cytoplasmic antibody (ANCA)-associated vasculitides (AAV) are a group of necrotizing multiorgan autoimmune vasculitides that predominantly affect small blood vessels and are associated with the presence of ANCAs. The main variants of AAV are microscopic polyangiitis (MPA), granulomatosis with polyangiitis (GPA) and eosinophilic granulomatosis with polyangiitis (EPA). Even though these entities have different clinical features, their immunological characteristics may overlap. Patients with AAV often develop rapidly progressive glomerulonephritis (RPGN) associated with focal necrotizing and crescentic lesions on renal biopsy and a lack of significant deposition of immunoglobulins in glomeruli (pauci-immune vasculitis). Besides renal involvement, AAV commonly affects lungs, upper respiratory tract and peripheral nervous system. Nevertheless, any organ may be involved [1,2,3]. The main subtypes of antineutrophil cytoplasmic antibodies (ANCA) that could be detected in AVV are perinuclear ANCAs (P-ANCA) and cytoplasmic ANCAs (C-ANCA). Indirect immunofluorescence specifies that the P-ANCA pattern is generally associated with myeloperoxidase (MPO) autoantibodies, while C-ANCAs target proteinase 3 (PR3) [4,5].

The precise mechanism of AAV development remains unknown. Nevertheless, the pathogenic role of ANCAs in AAV has been well-confirmed in in vitro analyses and in studies on animal models [3,6].

The role of T and B cells in AAV pathogenesis has been implicated. Aberrant functions of T and B lymphocytes, two major types of adaptive immunity cells, interfere with the host immune system and lead to tissue damage. Deregulation of T and B cells activates neutrophils and subsequently results in vascular inflammation and necrosis of the small blood vessels’ walls [7]. The pathogenic role of natural killer (NK) cells in AAV has also been suggested [8].

Considering the significant role of T, B and natural killer (NK) cells in the pathophysiology of AAV, the assessment of their distribution and disease-specific alterations could be helpful in establishing proper diagnosis [9]. So far, studies analyzing the AAV-related alterations in T, B and NK lymphocyte subsets distribution are sparse [8,10,11,12].

B cells are traditionally regarded as promoters of the immune response due to their capacity to produce antibodies and release a broad variety of cytokines. However, recent studies in B cell biology have also demonstrated that one functional B cell subset, regulatory B cells (Bregs), contributes to the maintenance of the proper immune balance. The most commonly used characterization of Breg cells was established by Blair et al. who identified CD19^+^CD24^++^CD38^++^ lymphocytes as B cells with regulatory properties [13]. Human B cells with regulatory functions have been described within the CD24^++^CD38^++^ transitional B cell subset [14]. IL-10 production is also considered to be a hallmark of Bregs [15]. Bregs restrain the excessive inflammatory responses that occur during autoimmune diseases or are caused by the exposure to various infectious factors [15]. Some of the activated B cells develop into extrafollicular plasmablasts and early memory B cells in the extrafollicular pathway [16]. Plasmablasts derived from the B cell lineage encompass a stage intermediate between activated B cells and plasma cells. Plasmablasts are generally rare in the peripheral blood of healthy individuals [17] and could be chronically elevated in persistent immune responses such as in chronic autoimmune diseases [18,19,20,21].

As previously mentioned, in addition to humoral immunity, cellular immunity is important in AAV pathogenesis and can be applied to multiple pathogenic mechanisms, including regulatory T cell (Treg CD4^+^CD25^+^FoxP3^+^) dysfunction and imbalance of certain T cell subsets. Treg cells are a specialized T cell subpopulation capable of controlling and limiting the harmful immune response. Tregs also prevent the immune response against self-antigens and have a strong suppressive activity under inflammatory conditions [22]. Regulatory T cells subpopulation contributes markedly to the maintenance of peripheral tolerance through the mechanism of controlling circulating autoreactive T cells [23]. Th17 cells, a subpopulation of T cells identified by their ability to produce IL-17, are involved in the early response to extracellular pathogens and appear to significantly interfere with long-lasting inflammatory responses in chronic infections and autoimmune disorders. In a mouse model, individuals lacking the gene for IL-17 were more susceptible to infection and had disturbances in renal-dependent IL-17 migration of neutrophils to the site of infection [24]. Furthermore, it was observed that IL-17 induces neutrophil migration to the lung in a mouse model of acute airway inflammation induced by *Mycoplasma pneumoniae* [25]. Another effector memory cells population, CD4 T cells, that do not express the costimulatory molecule CD28 (CD4^+^CD28^−^) has acquired a cytotoxic phenotype with the expression of perforin and granzyme B and has been shown to be cytotoxic to endothelial cells and induce vascular injury [26,27]. CD4^+^CD28^−^ T cells have proinflammatory properties and their proportions are known to be upregulated in several inflammatory disorders [28,29,30]. These T cells may have a role in accelerating the atherosclerotic process and/or destabilization of plaques in patients with AAV [31,32].

Currently, the most widely used reliable and validated tool for assessing disease activity and the extent of the disease in AAV is the Birmingham Vasculitis Activity Score (BVAS). BVAS is a clinical checklist of relevant signs, symptoms and features of active AAV that provides a standardized measure of disease severity and enables introducing proper treatment. Because of the fact that a specific biomarker that can quantify disease activity is lacking, a validated tool like BVAS is needed in clinical practice [33]. The presence of ANCAs in the patient’s serum strongly suggests AAV, and the elevated level of these antibodies may reflect active disease. Nevertheless, ANCA titers should not be considered a reliable marker of disease activity [34,35]. Though ANCA titers could be useful in monitoring the course of disease, the utility of their serial measurements to predict disease relapse remains controversial [35,36,37]. There are several factors that could predict the exacerbation of the disease; nevertheless, a specific factor of AAV relapse has not been established yet [38,39,40]. At present, specific markers of disease activity in AAV are lacking, thus the search for reliable biomarkers of disease activity is needed.

One of the most promising markers of immune activation in the AAV course is the suPAR (soluble urokinase plasminogen activator receptor). SuPAR is a circulating form of a glycosylphosphatidylinositol-linked membrane protein that is expressed on different cells including neutrophils, phagocytes, endothelial cells and activated T cells [41], most of which are involved in the pathogenesis of AAV. The suPAR level reflects the activation state of the immune system and has already been considered a promising biomarker in a number of immune-based diseases, such as cardiovascular disease, lupus nephritis (LN), rheumatoid arthritis (RA) or focal segmental glomerulosclerosis [42,43,44,45].

The aim of the study was to assess regulatory and effector cell populations accompanied by the suPAR biomarker level and link the so-defined immune state to the AAV disease activity and clinical manifestations. The multicomponent description of the immune state in AAV might aid in defining immune hallmarks of upcoming exacerbation of the disease.

## 2. Materials and Methods

A group of 56 ANCA-positive patients with AAV (29 female patients, 27 male patients, mean age: 56.6 ± 16.4 years) were enrolled in this study. The patients attending or referred to the Department of Nephrology and Transplantation Medicine at the University Hospital were consecutively included between September 2017 and January 2020. The Chapel Hill Consensus Conference (CHCC) nomenclature was used to define GPA and MPA [46]. The diagnosis of GPA was based on the American College of Rheumatology (ACR) classification, while the validated stepwise classification algorithm proposed by Watts et al. was used to classify patients as suffering from GPA or MPA [47,48]. According to this classification algorithm, 40 patients met the criteria for GPA, 16 individuals—for MPA (Table 1).

All the patients included in this study had renal involvement; moreover, some patients had lung (*n* = 37), joint (*n* = 9), ear (*n* = 9), gastrointestinal tract (*n* = 7), central nervous system (*n* = 7), upper respiratory tract (*n* = 7), skin (*n* = 2) and/or eye (*n* = 1) involvement.

Two patients did not receive any AAV-directed drugs and 11 patients received corticosteroids monotherapy at the time of sampling. The remaining 43 patients received corticosteroids in combination with immunosuppressive therapy including cyclophosphamide (19), mycophenolate mofetil (12), azathioprine (9) or methotrexate (3). Nine patients at the time of sampling presented activation of the disease requiring intensification of the immunosuppressive therapy, and five were considered for tapering of drug therapy. None of the participants was treated with rituximab at the time of sampling.

Patients with a known malignancy, ongoing infection or coexisting autoimmune disorder were excluded from the study.

The assessment of vasculitis disease activity was performed using the BVAS according to the European League Against Rheumatism recommendations.

Control samples were obtained from 20 healthy age- and sex-matched subjects (HC). The study was approved by the Wroclaw Medical University Bioethics Committee and informed consent was obtained from each participant.

### 2.1. Flow Cytometry

The following mouse anti-human antibodies (BD) were used for cell phenotyping: anti-CD3-APC (clone UCHT1), anti-CD3-PerCP (clone SK7), anti-CD4-PerCP (clone SK3), anti-CD8-FITC (clone RPA-T8), anti-CD19-PE (clone HIB19), anti-CD24-PerCP-Cy 5.5 (clone ML5), anti-CD25-FITC (clone M-A251), anti-CD27-APC (clone M-T271), anti-CD28-PE (clone CD28.2), anti-CD38-FITC (clone HIT2), anti-CD57-FITC (clone HNK-1), PE Mouse Anti-Human FoxP3 (clone: 259D/C7), anti-IL-17A Alexa Fluor 647 (clone N49-653), anti-IL-17F Alexa Fluor 647 (clone O33-782) and the four-color BD Multitest (CD3/CD16+CD56/CD45/ CD19) with Trucount tubes.

The absolute numbers of T, B, NK and NKT cells per µL of blood were determined with the BD Multitest according to the manufacturer’s protocol. All the T or B subpopulations were counted in relation to T or B cells, which also enabled their enumeration per µL of blood.

The Treg population was assessed through CD3^+^CD4^+^CD25^+^FOXP3^+^ phenotyping. Heparinized blood (300 µL) was stained with 20 µL of the following antibodies: anti-CD4-PerCP, anti-CD3-APC and anti-CD25-FITC. After incubation, the red blood cells were lysed with a BD FACS Lysing Solution (BD). The cells were washed with phosphate-buffered saline supplemented with 2% fetal bovine serum (PBS-FBS) and processed with a Human FoxP3 Buffer Set (BD) according to manufacturer instructions. The cells were stained with 10 µL of the anti-FoxP3-PE antibody for 30 min at 4 °C in the dark. The samples were then washed twice with PBS-FBS.

The T CD4^+^CD28^−^ cells subpopulation was enumerated in 300 µL of heparinized blood samples stained with 20 µL of the following antibodies: anti-CD3-APC, anti-CD4-PerCP, anti-CD57-FITC and anti-CD28-PE. After 30 min of incubation, the red blood cells were lysed with a BD FACS Lysing Solution (BD). The cells were washed twice with PBS-FBS and analyzed by means of flow cytometry.

The counts of Th17 and Tc17 were determined by intracellular staining of 300 µL of heparinized blood samples with the following antibodies: anti-CD3-PerCP, anti-CD8-FITC, anti-IL-17A Alexa Fluor 647 and anti-IL-17F Alexa Fluor 647. Th17a and Th17f were enumerated by assessment of the intracellular expression of IL-17A and IL-17F. Prior activation of the whole blood samples with PMA and ionomycin was necessary to visualize the expression of IL-17 of the Th17 cells. As long as PMA/ionomycin stimulation can cause downregulation of CD4 expression on the cell surface, the number of Th17 cells was assessed in the CD3^+^CD8^−^ subpopulation.

Subpopulations of B cells were analyzed with CD19, CD24, CD27 and CD38 phenotyping according to de Masson et al. [14]. We identified four main B cell subsets: immature transitional B cells (CD19^+^CD24^++^CD38^++^), naïve B cells (CD19^+^CD24^INT^CD38^INT^) that had not encountered an antigen, memory B cells (CD19^+^CD27^+^) and plasmablasts (CD19^+^CD24^−^CD38^++^CD27^++^). Heparinized blood (300 µL) was stained with the following antibodies: anti-CD19-PE, anti-CD27-APC, anti-CD38-FITC and anti-CD24-PerCP-Cy 5.5. After 30 min of incubation, the red blood cells were lysed with a BD FACS Lysing Solution (BD). The cells were washed twice with PBS-FBS and analyzed by means of flow cytometry.

**Figure 1:**
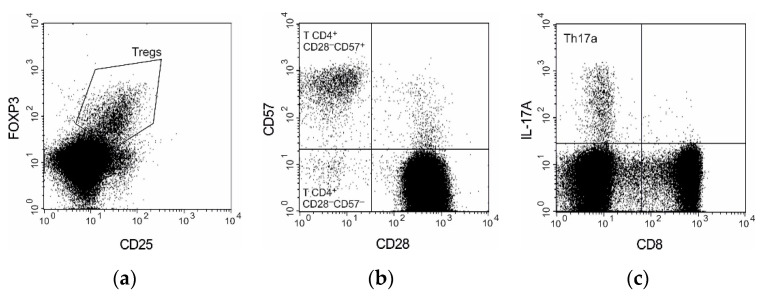
T subpopulations gating strategy. (**a**) Regulatory T cells gated on CD4^+^ T lymphocytes; (**b**) CD28-negative subpopulations gated on CD4^+^ T lymphocytes; (**c**) Th17a cells gated on T lymphocytes.

**Figure 2:**
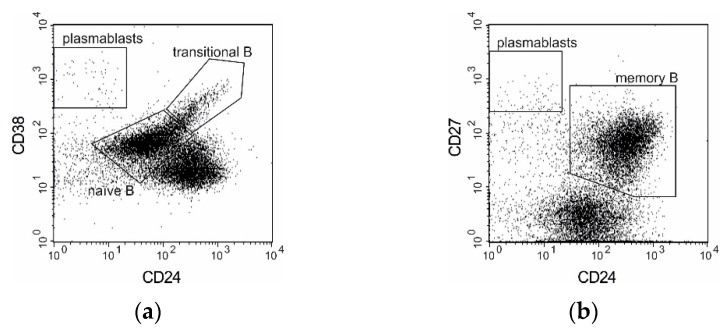
B subpopulations gating strategy. Dot plots generated for CD19^+^ lymphocytes. (**a**) Gating of transitional and naïve B cells; (**b**) gating of memory B cells; plasmablasts defined as the intersection of gates from both panels.

The gating strategies used for analysis and enumeration of T and B cells subpopulations are presented on Figure 1 and Figure 2, respectively.

### 2.2. ELISA Assay

The suPAR concentration was determined in the EDTA–plasma samples with the suPARnostic ELISA assay according to the manufacturer’s instructions (Virogates, Denmark).

### 2.3. Statistical Analysis

Descriptive statistics were calculated for all the demographics, clinical characteristics and laboratory data. All the presented comparisons and correlations included nonnormally distributed variables. Intergroup comparisons of continuous data were assessed using the nonparametric Mann–Whitney U test. The correlations were performed using rank correlation (Spearman’s). The statistical test results, for which the *p*-values were lower than 0.05, were considered significant. Statistical analysis was performed using the Statistica (version 13.3; StatSoft) software. 

## 3. Results

### 3.1. Flow Cytometry—General Lymphocytes Phenotyping

The results of the general lymphocytes phenotyping in the AAV patients and the heathy controls are summarized in Table 2. We did not observe any statistically significant difference in absolute counts of T lymphocytes between the AAV patients and the controls. NK (134, 89–198 vs. 227, 164–330 cells/µL) and NK^bright^ cells (8, 5–15 vs. 21, 15–27 cells/µL) counts were statistically significantly lower in the AAV patients than in the controls (*p* < 0.001). Furthermore, the B lymphocyte population was slightly decreased in the AAV patients compared to the HC (160, 65–254 vs. 211, 169–376 cells/µL, *p* = 0.035). There were no significant differences in the absolute counts of the general lymphocytes populations between the GPA and MPA patients (Table 3).

### 3.2. T Cell Subpopulations

**Figure 3:**
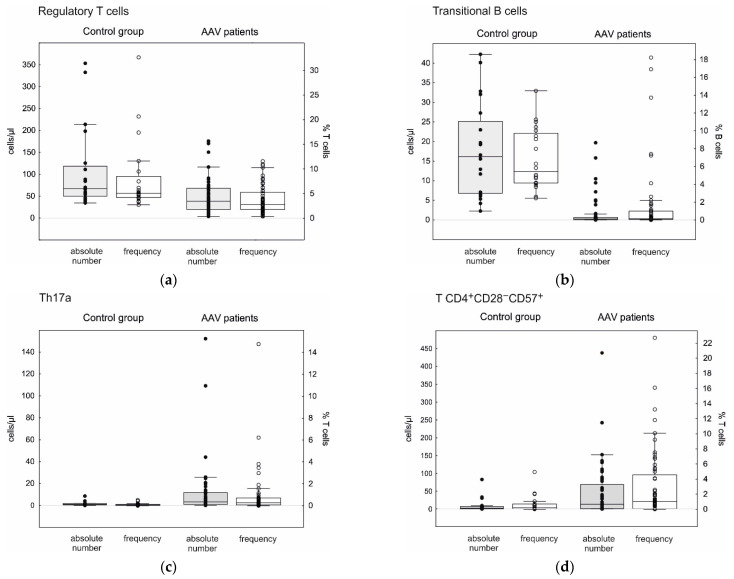
Absolute counts and frequency of selected peripheral blood lymphocyte subpopulations. Box—IQR, line—median, whiskers—non-outliers range, ○—raw frequency data, ●—raw absolute count data. (**a**) Regulatory T cells: absolute count, AAV vs. HC—40, 19–71 cells/µL vs. 67, 50–118 cells/µL, *p* < 0.001; frequency, AAV vs. HC—2.8, 1.8–5.5% vs. 5.1, 4.2–8.5% T lymphocytes, *p* < 0.001. (**b**) Transitional B cells: absolute count, AAV vs. HC—0.3, 0.1–0.7 cells/µL vs. 16.1, 6.8–25.1 cells/µL, *p* < 0.001; frequency, AAV vs. HC—0.2, 0.1–1.0% vs. 5.4, 4.2–9.7% B lymphocytes, *p* < 0.001. (**c**) Th17a: absolute count, AAV vs. HC—3.3, 0.7–11.7 cells/µL vs. 0.7, 0.4–1.8 cells/µL, *p* < 0.001; frequency, AAV vs. HC—0.29, 0.08–0.74% vs. 0.05, 0.03–0.13% T lymphocytes, *p* < 0.001. (**d**) T CD4^+^CD28^−^CD57^+^: absolute count, AAV vs. HC—13.5, 1.1–69.1 cells/µL vs. 2.5, 0.6–7.6 cells/µL, *p* = 0.023; frequency, AAV vs. HC—1.1, 0.3–4.6% vs. 0.20, 0.05–0.68% T lymphocytes, *p* = 0.010.

We observed AAV-related changes in cell profiles of the peripheral blood T lymphocytes (Table 4, Figure 3). The absolute counts of Th17a and Th17f cells were significantly higher in the AAV patients compared to the healthy controls (3.3, 0.8–11.7 cells/µL vs. 0.7, 0.4–1.8 cells/µL, *p* < 0.001, and 0.5, 0.1–1.2 cells/µL vs. 0.1, 0.0–0.3 cells/µL, *p* < 0.001, respectively).

We also observed an expansion of CD28-negative cells within the peripheral blood CD4^+^ population in the AAV patients compared to the healthy controls, both as CD57-positive (13.5, 1.1–69.1 cells/µL vs. 2.5, 0.6–7.6 cells/µL, *p* = 0.023) or CD57-negative ones (4.6, 1.0–12.3 cells/µL vs. 0.7, 0.3–2.6 cells/µL, *p* = 0.016).

On the contrary, the Treg population was deeply reduced in the AAV patients compared to the HC (40, 19–71 cells/µL vs. 67, 50–118 cells/µL, *p* = 0.001).

We did not observe any differences in cell profiles of the peripheral blood T lymphocytes between the MPA and GPA patients (Table 5).

**Table 4:** T and B cell subsets in the AAV patients and the healthy controls.

Cell Population		AAV Patients		Healthy Controls		*p*-Value
		Mean ± SD	Median, IQ Range	Mean ± SD	Median, IQ Range	
Th17a cells	Cells/µL	11.23 ± 25.10	3.3, 0.8–11.7	1.42 ± 1.95	0.7, 0.4–1.8	<0.001
	% T	1.117 ± 2.675	0.29, 0.08–0.74	0.108 ± 0.134	0.05, 0.03–0.13	<0.001
Th17f cells	Cells/µL	1.11 ± 1.79	0.5, 0.1–1.2	0.53 ± 1.43	0.1, 0.0–0.3	<0.001
	% T	0.279 ± 1.364	0.04, 0.01–0.07	0.043 ± 0.123	0.01, 0.00–0.02	<0.001
Treg cells	Cells/µL	55.5 ± 61.8	40, 19–71	107.3 ± 94.3	67, 50–118	0.001
	% T	9.19 ± 34.29	2.8, 1.8–5.5	8.16 ± 7.43	5.1, 4.2–8.5	<0.001
T CD4^+^CD28^−^CD57^+^	Cells/µL	44.78 ± 74.25	13.5, 1.1–69.1	10.03 ± 19.60	2.5, 0.6–7.6	0.023
	% T	3.09 ± 4.56	1.1, 0.3–4.6	0.69 ± 1.18	0.20, 0.05–0.68	0.010
T CD4^+^CD28^−^CD57^−^	Cells/µL	12.29 ± 24.87	4.6, 1.0–12.3	80.63 ± 243.95	0.7, 0.3–2.6	0.016
	% T	0.95 ± 1.73	0.4, 0.1–1.1	5.58 ± 16.83	0.07, 0.02–0.16	0.013
Plasmablasts	Cells/µL	2.27 ± 5.58	0.8, 0.4–1.9	1.06 ± 0.69	1.0, 0.6–1.3	0.773
	% B	5.51 ± 22.10	0.6, 0.2–1.3	0.53 ± 0.41	0.4, 0.2–0.8	0.222
Naïve B cells	Cells/µL	114.9 ± 133.7	72, 27–153	153.4 ± 94.5	121, 80–227	0.017
	% B	49.0 ± 21.4	55, 30–67	56.9 ± 11.1	55, 46–67	0.339
Transitional B cells	Cells/µL	1.66 ± 3.85	0.3, 0.1–0.7	18.00 ± 11.88	16.1, 6.8–25.1	<0.001
	% B	1.59 ± 3.86	0.2, 0.1–1.0	6.81 ± 3.40	5.4, 4.2–9.7	<0.001
Memory B cells	Cells/µL	59.6 ± 53.4	44, 17–90	73.7 ± 48.4	60, 44–97	0.110
	% B	37.8 ± 24.4	29, 19–56	29.4 ± 11.5	30, 21–37	0.544

### 3.3. B Cell Subpopulations

We also analyzed AAV-related changes in cell profiles of the peripheral blood B cells (Table 4, Figure 3). We observed a significantly lower count of the CD19^+^CD24^++^CD38^++^ transitional B cells in the AAV patients compared to the HC (0.3, 0.1–0.7 cells/µL vs. 16.1, 6.8–25.1 cells/µL, *p* < 0.001). Additionally, the number of CD19^+^CD24^int^CD38^int^ naïve B cells was slightly lower in the AAV patients compared to the healthy controls (72, 27–153 cells/µL vs. 121, 80–227 cells/µL, *p* = 0.017).

We did not observe any difference in the memory CD19^+^CD27^+^ nor CD19^+^CD24^−^CD38^++^CD27^++^ plasmablast cell counts between the healthy control group and the AAV patients. Furthermore, there was no difference in B cell profiles between the GPA and MPA patients.

### 3.4. Lymphocyte Subpopulations and AAV Disease Activity

**Figure 4:**
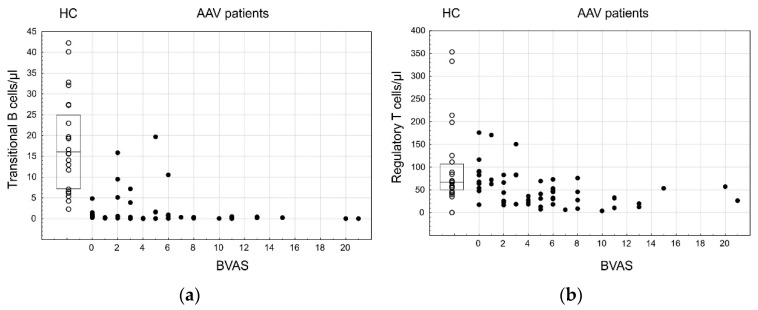
Transitional B cells and regulatory T cells and their relation to the AAV disease and its activity; ○—HC, box—IQR, line—median, ●—AAV. (**a**) Transitional B cells—HC vs. AAV, *p* < 0.001; correlation with the BVAS, rs = −0.35, *p* = 0.009. (**b**) Regulatory T cells ― HC vs. AAV, *p* < 0.001; correlation with the BVAS, rs = −0.43, *p* < 0.001.

The general overview of the peripheral blood lymphocytes provided an AAV-related immune fingerprint characterized by an expansion of Th17 cells and T cells lacking the costimulatory molecule CD28 accompanied by a decrease of regulatory populations (Tregs and transitional B cells) and NK cells. We analyzed the relationship between those populations and the AAV disease activity (Table 6) and observed that, among them, only Tregs and transitional B cells were negatively associated with the BVAS (rs = −0.43, *p* < 0.001, and rs = −0.35, *p* = 0.009, respectively; Figure 4).

### 3.5. SuPAR

**Figure 5:**
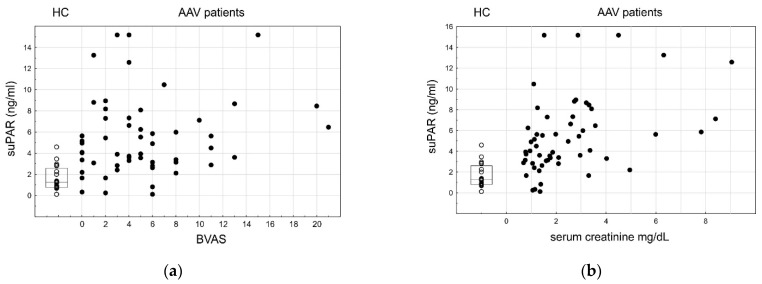
SuPAR plasma level and its relation to the AAV disease (suPAR HC vs. AAV, *p* < 0.001), its activity and the serum creatinin concentration; ○—HC, box—IQR, line—median, ●—AAV. (**a**) Correlation of the suPAR plasma level in AAV with the BVAS, rs = 0.11, *p* = 0.406. (**b**) Correlation of the suPAR plasma level in AAV with serum creatinine, rs = 0.43, *p* < 0.001.

The SuPAR plasma concentration was higher in the patients than in the healthy controls (median, IQR: 4.7, 3.1–7.2 ng/mL vs. 1.3, 0.8–2.5 ng/mL, *p* < 0.001). Its level did not vary between the GPA and MPA patients (*p* = 0.369). Despite the AAV-related increase of the suPAR, its plasma level was not proved to be related to the overall disease activity (BVAS, rs = 0.11, *p* = 0.406, Figure 5). We did not find any significant correlation between the suPAR level and the lymphocyte subpopulations in the AAV individuals. However, we observed the statistically significant correlation between the suPAR plasma level and the kidney function (serum creatinine level, rs = 0.43, *p* < 0.001), showing that deterioration of the kidney function in AAV with renal involvement is associated with the immune activation state as defined by the suPAR level.

## 4. Discussion

We performed an overview of peripheral blood lymphocytes patterns in AAV patients. Our results provided an AAV-related immune fingerprint characterized by an expansion of Th17 cells and T cells lacking the costimulatory molecule CD28 accompanied by a decrease of regulatory populations (Tregs and transitional B cells) and NK cells. We also observed that despite the strong association of those populations with AAV diagnosis, only Tregs and transitional B cells presented significant relation to the disease activity in our group of patients.

Despite the low number of studies, early studies in the 1990s reported contradictory findings in terms of T lymphocyte numbers in AAV patients. Published in 1995, a study by Schlesier et al. reported that the CD4^+^ subset was significantly diminished, while the percentage of CD8^+^ T cells was elevated in GPA patients as compared to HC [49]. However, Moosig et al. showed no differences in the numbers of these cells [50]. In our study, there were no significant differences in the general T lymphocyte numbers between the AAV patients and the healthy controls.

Immune disturbances in AAV may be associated with abnormal homeostasis or defective function of regulatory cells, which has been proved in a number of studies addressing the size and activity of this subpopulation in patients with AAV. A vast majority of investigators has indicated a reduced number of circulating Treg cells in AAV [51,52,53,54,55] and negative correlation with disease activity [56,57]. Contrarily, Free et al. noted significantly increased Treg cell frequencies in patients with active disease but suggested their decreased suppressive function [58]. Our results showed a profound decline in the number of Tregs in the AAV patients and their relation to disease activity.

It is noteworthy that in our study, the size of the Treg population of the AAV patients with low disease activity resembled that of the healthy controls.

There is a lot of clinical and experimental evidence that Th17 cells can induce inflammation of tissues in several autoimmune diseases, including pathogenesis of AAV. There are publications reporting AAV patients with an increased concentration of IL-17 in serum and Th17 cell expansion in peripheral blood [59,60,61]. Wang et al. verified that both the percentages and the absolute numbers of Th17 cells were elevated in AAV patients compared with HCs, although the differences were not statistically significant. In addition, there were no significant differences in the Th17 cells frequency and numbers between the active stage and the remission [57]. Our results indicated a significant expansion of Th17a and Th17f cell populations in the AAV patients compared to the healthy controls; however, their potential relation to disease activity was not proved.

Taken together, those observations suggested that the changes in Th17 and Treg levels are likely to play pathological roles in renal vasculitis patients. Furthermore, patients with renal vasculitis displayed an increased proportion of Th17 cells and a decreased proportion of Treg cells. This may suggest considerable association with renal organ involvement. The imbalance of Th17 and Treg cells can also be used potentially as a biomarker to monitor AAV patients with kidney involvement.

A subset of circulating T cells lacking the costimulatory molecule CD28 is expanded in GPA patients and displays potent effector functions [49,50,62]. Loss of the costimulatory molecule CD28 on CD4^+^ T cells suggests repeated exposure to a persistent antigen [30]. The cells devoid of the CD28 molecule might contribute to disease progression and autoreactivity either directly, by maintaining the inflammatory response, or as a result of ongoing activation. Peripheral blood CD4^+^CD28^−^ T cells and CD4^+^CD28^−^ T cells within granulomatous lesions are a major source of Th1-type cytokine secretion which is mainly restricted to TNFα and IFNγ [63]. Oligoclonality and shortened telomers suggest clonal expansion and replicative senescence of T cells in ANCA-associated vasculitides [64,65]. Lack of regulatory control over the senescent T cells and deterioration of the regulatory T cells number might be factors associated either with initial antigen-driven incidents or at least contributing to chronic inflammation and autoreactivity in ANCA-associated vasculitides. We observed a greater expansion of the CD28^−^ cells within the CD4^+^ population in peripheral blood of the AAV patients compared to the healthy controls, but their number was not related to disease activity.

The role of B cells in the pathogenesis of AAV is well-established, as confirmed by the effectiveness of the therapy involving B cells depletion [66,67]. Development of B cells in germinal centers is reflected by differential expression of a variety of B cell surface antigens, such as surface Ig, CD38, CD20, CD27 and CD24. There is a relationship between the expression of each of the differentiation clusters and B cell maturation [14]. A separate subset of interleukin IL-10-producing B cells (Breg) regulating T cell-mediated immunity has been identified [68]. Early studies in the 1990s reported that both active and inactive patients have similar circulating B cell levels compared to HC [50]. There are sparse reports indicating that the number of regulatory B cells is reduced in patients with AAV [69,70,71,72].

Transitional B cells (CD19^+^CD24^++^CD38^++^) are a link between immature B cells in the bone marrow and mature peripheral B cells. They represent one of the regulatory B cell subpopulations, and their frequency in circulation may be altered in autoimmune diseases, such as multiple sclerosis, neuromyelitis optica spectrum disorders, systemic lupus erythematosus, Sjögren syndrome, rheumatoid arthritis, systemic sclerosis and juvenile dermatomyositis [73]. Our results suggest the potential role of the transitional B cells population in the prevention of AAV disease activation. They are in line with those findings which suggest that regulatory B cells contribute to the maintenance of the balance required for tolerance and prohibit ANCA production in AAV.

Breg cells have also been shown to suppress T cell proliferation and T cell-dependent inflammatory function [74,75]. Moreover, it has been demonstrated that CD19^+^CD24^++^CD38^++^ Breg cells support regulatory T cells while limiting Th1 and Th17 differentiation [75]. Von Borstel et al. observed that CD19^+^CD24^++^CD38^++^ Bregs were inversely correlated with Th17 effector memory cells (CD3^+^CD4^+^CD45RO^+^CCR7^−^CCR6^+^ CXCR3^−^CCR4^+^) in GPA patients in remission. Moreover, the authors discovered that CD19^+^CD24^++^CD38^++^ Bregs seem to suppress Th17 cell expansion in vitro [76]. Our findings are in line with those data since a decreased number of Treg cells and an increased number of Th17 cells in the AAV patients were observed.

A study by Tognarelli et al. showed that NK cells from GPA patients expressed markers of recent in vivo activation, degranulated more efficiently than control NK cells in the presence of renal MECs and induced direct killing of renal MECs in vitro. The authors suggested that in inflammatory conditions in GPA, renal MECs may contribute to the recruitment and activation of NK cells in the target vessel walls, which may participate in the necrotizing vasculitis of the kidney during disease [77]. Merkt et al. showed an inverse correlation of NK cell numbers with GPA activity. The authors suggested that reduced CD56 NK cells in active GPA have an activated phenotype and play a role in the pathogenesis and/or modulation of inflammation in GPA [8]. In the present study, we also observed a slightly decreased population of NK cells in the AAV patients; however, their association with disease activity was not observed.

In a number of studies, it has been suggested that the suPAR level reflects the activation state of the immune system [42,43,44,45]. There are a few studies suggesting that the suPAR plasma level can be considered a biomarker in AAV as well [78]. The results of our study confirm that an elevated level of the suPAR is associated with the AAV disease, and that its increased level is related to AAV-related deterioration of the kidney function.

To conclude, our results indicate that AAV is associated with alterations in lymphocyte subpopulations, but these alterations are rather linked to the activity state of the disease rather than its classification variant. We believe that further investigations in larger groups of patients are required to fully elucidate the mechanisms involved in the pathogenesis of the disease and the role of individual subpopulations in the AAV pathogenesis, progression and clinical manifestations.

### Limitations of the Study

Our results are from a single-center study and include individuals with two major variants of AAV, granulomatosis with polyangiitis and microscopic polyangiitis, which, although different in clinical features, present overlapping immunological characteristics. As our study group included a limited number of participating patients, we did not observe immunological differences between the variants. Further research on a larger cohort of patients would be valuable to support the conclusions.

## Figures and Tables

**Table 1:** The patients’ clinical characteristics.

Patients’ Characteristics	GPA Patients		MPA Patients	
*N*	40		16	
Sex (M/F)	18/22		9/7	
ANCA-positive/negative	33/7		16/0	
	Mean ± SD	Median, IQR	Mean ± SD	Median, IQR
Age (years)	54.6 ± 17.8	58, 43–66	61.8 ± 10.8	62, 59–69
MPO-ANCA, RU/ml	130.9 ± 235.9	40, 13–119	–	–
PR3-ANCA, RU/ml	–	–	83.0 ± 43.6	80, 64–117
Creatinine	1.91 ± 1.24	1.4, 1.1–2.4	3.92 ± 2.53	3.1, 2.5–5.0
CRP	29.06 ± 32.53	16.8, 3.5–46	12.1 ± 19.7	4.3, 2.7–7.2
BVAS	5.5 ± 4.7	5, 0–20	4.3 ± 5.1	4, 0–21

**Table 2:** General lymphocytes populations in the AAV patients and the healthy controls.

Cell Population Cells/µL	AAV Patients		Healthy Controls		*p*-Value
	Mean ± SD	Median, IQ Range	Mean ± SD	Median, IQ Range	
Total lymphocytes	1766.0 ± 775.9	1725, 1244–2176	1870.3 ± 434.3	1821, 1504–2222	0.219
T lymphocytes	1336.4 ± 609.2	1316, 946–1678	1338.7 ± 349.9	1267, 1113–1680	0.686
CD8^+^ T lymphocytes	492.5 ± 277.1	409, 263–691	500.7 ± 205.1	514, 326–658	0.703
CD4^+^ T lymphocytes	854.8 ± 442.6	836, 560–1061	826.1 ± 223.4	847, 668–982	0.949
CD4/CD8	2.06 ± 1.22	1.7, 1.2–2.6	1.86 ± 0.76	1.7, 1.4–2.1	0.925
B lymphocytes	198.2 ± 176.5	160, 65–254	261.5 ± 133.6	211, 169–376	0.035
NK cells	177.3 ± 176.3	134, 89–198	252.5 ± 106.4	227, 164–330	<0.001
NKT cells	121.9 ± 116.9	88, 47–154	105.5 ± 70.8	94, 54–138	0.995
NK^bright^ cells	10.0 ± 7.5	8, 5–15	22.0 ± 9.7	21, 15–27	<0.001

**Table 3:** General lymphocytes populations in the GPA and MPA patients.

Cell Population Cells/µL	GPA Patients		MPA Patients		*p*-Value
	Mean	Median, IQ Range	Mean	Median, IQ Range	
Total lymphocytes	1904.9 ± 824.7	1809, 1307–2359	1419.6 ± 509.6	1505, 969–1801	0.050
T lymphocytes	1414.5 ± 661.1	1334, 975–1790	1140.9 ± 409.0	1231, 785–1441	0.140
CD8^+^ T lymphocytes	538.6 ± 296.9	472, 301–733	377.4 ± 180.7	344, 246–564	0.050
CD4^+^ T lymphocytes	897.2 ± 489.6	885, 565–1115	748.6 ± 279.6	738, 524–967	0.363
CD4/CD8	1.99 ± 1.32	1.5, 1.1–2.4	2.25 ± 0.93	2.1, 1.7–3.0	0.144
B lymphocytes	220.2 ± 190.4	188, 85–264	143.9 ± 123.6	101, 51–239	0.178
NK lymphocytes	199.3 ± 199.5	155, 97–212	122.3 ± 77.4	112, 78–186	0.197
NK^bright^ lymphocytes	10.3 ± 8.17	8, 5–15	9.31 ± 5.66	8, 5–15	0.847

**Table 5:** T and B cell subsets in the GPA and MPA patients.

Cell Population		GPA Patients		MPA Patients		*p*-Value
		Mean	Median, IQ Range	Mean	Median, IQ Range	
Th17a cells	Cells/µL	13.41 ± 28.77	4.7, 0.8–13.6	5.79 ± 10.68	2.3, 0.8–5.6	0.314
	% T	1.379 ± 3.105	0.30, 0.08–0.93	0.461 ± 0.726	0.22, 0.09–0.51	0.508
Th17f cells	Cells/µL	1.34 ± 2.05	0.6, 0.2–1.5	0.54 ± 0.63	0.3, 0.1–0.7	0.145
	% T	0.370 ± 1.610	0.05, 0.01–0.09	0.053 ± 0.072	0.02, 0.01–0.06	0.257
Treg cells	Cells/µL	58.4 ± 67.6	47, 20–71	48.4 ± 44.9	27, 20–68	0.571
	% T	11.24 ± 40.49	2.8, 1.8–5.9	4.09 ± 3.26	3.3, 1.9–4.7	0.942
T CD4^+^CD28^−^CD57^+^	Cells/µL	38.88 ± 47.76	14.0, 1.1–81.1	59.54 ± 118.14	8.8, 2.3–58.8	0.596
	% T	2.80 ± 3.58	1.0, 0.3–5.1	3.82 ± 6.49	1.2, 0.2–4.1	0.828
T CD4^+^CD28^−^CD57^−^	Cells/µL	12.7 ± 28.0	5, 2–12	11.2 ± 15.2	3, 0.3–20	0.686
	% T	1.03 ± 1.98	0.4, 0.2–1.0	0.76 ± 0.90	0.4, 0.0–1.5	0.618
Naïve B cells	Cells/µL	133.4 ± 147.4	100, 26–159	68.8 ± 76.7	45, 27–89	0.145
	% B	50.7 ± 22.2	58, 29–68	44.7 ± 19.3	46, 34–58	0.235
Memory B cells	Cells/µL	59.9 ± 44.2	57, 21–95	58.8 ± 73.2	23, 15–77	0.344
	% B	37.0 ± 25.1	28, 18–58	39.6 ± 23.3	31, 20–56	0.496
Transitional B cells	Cells/µL	1.61 ± 3.66	0.3, 0.1–0.9	1.77 ± 4.42	0.1, 0.1–0.6	0.145
	% B	1.25 ± 2.66	0.2, 0.1–1.1	2.43 ± 5.93	0.1, 0.0–0.5	0.502
Plasmablasts	Cells/µL	2.89 ± 6.51	1.0, 0.4–2.5	0.71 ± 0.47	0.1, 0.4–1.0	0.184
	% B	7.09 ± 26.00	0.5, 0.2–1.3	1.54 ± 2.82	0.9, 0.2–1.3	0.827

**Table 6:** Relationship between the AAV-related immune fingerprint cell populations and the disease activity in the patients group.

Cell Population		Association with the BVAS	
		Correlation Coefficient, rs	*p*-Value
Th17a cells	Cells/µL	−0.17	0.213
	% T	−0.18	0.190
Th17f cells	Cells/µL	−0.23	0.094
	% T	−0.18	0.188
Treg cells	Cells/µL	−0.43	< 0.001
	% T	−0.36	0.009
T CD4^+^CD28^−^CD57^+^	Cells/µL	−0.07	0.588
	% T	−0.03	0.800
T CD4^+^CD28^−^CD57^−^	Cells/µL	0.05	0.712
	% T	0.10	0.467
Transitional B cells	Cells/µL	−0.35	0.009
	% B	−0.23	0.089
NK cells	Cells/µL	−0.19	0.159
NK^bright^ cells	Cells/µL	0.00	0.959

## Data Availability

The data presented in this study are available upon request from the corresponding author.
